# Numbers in Context: Cardinals, Ordinals, and Nominals in American English

**DOI:** 10.1111/cogs.13471

**Published:** 2024-06-19

**Authors:** Greg Woodin, Bodo Winter

**Affiliations:** ^1^ Department of English Language and Linguistics University of Birmingham

**Keywords:** Numerical cognition, Mathematical cognition, Corpus linguistics, Mental number line, Rounding, Approximate number system, Register studies

## Abstract

There are three main types of number used in modern, industrialized societies. Cardinals count sets (e.g., people, objects) and quantify elements of conventional scales (e.g., money, distance), ordinals index positions in ordered sequences (e.g., years, pages), and nominals serve as unique identifiers (e.g., telephone numbers, player numbers). Many studies that have cited number frequencies in support of claims about numerical cognition and mathematical cognition hinge on the assumption that most numbers analyzed are cardinal. This paper is the first to investigate the relative frequencies of different number types, presenting a corpus analysis of morphologically unmarked numbers (not, e.g., “eighth” or “21st”) in which we manually annotated 3,600 concordances in the Corpus of Contemporary American English. Overall, cardinals are dominant—both pure cardinals (sets) and measurements (scales)—except in the range 1,000–10,000, which is dominated by ordinal years, like 1996 and 2004. Ordinals occur less often overall, and nominals even less so. Only for cardinals do round numbers, associated with approximation, dominate overall and increase with magnitude. In comparison with other registers, academic writing contains a lower proportion of measurements as well as a higher proportion of ordinals and, to some extent, nominals. In writing, pure cardinals and measurements are usually represented as number words, but measurements—especially larger, unround ones—are more likely to be numerals. Ordinals and nominals are mostly represented as numerals. Altogether, this paper reveals how numbers are used in American English, establishing an initial baseline for any analyses of number frequencies and shedding new light on the cognitive and psychological study of number.

## 1 Introduction

**Fig. 1:**
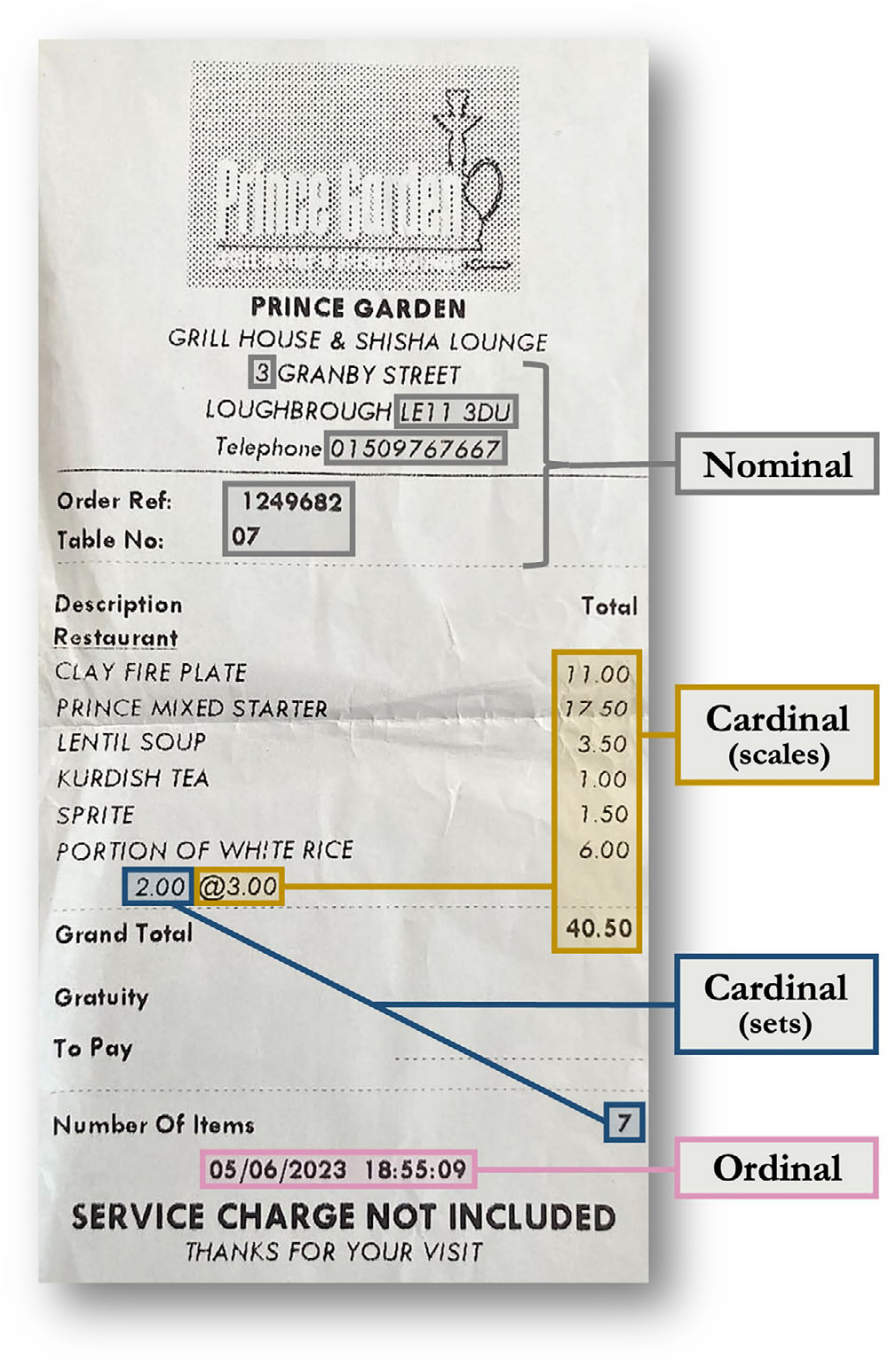
A bill from a restaurant evidencing different number types, which are annotated and color‐coded—blue: cardinals that count sets; yellow: cardinals that quantify elements of conventional scales; pink: ordinals that identify positions in an ordered sequence; and gray: nominals that serve as unique identifiers.

Imagine you are in a restaurant and your server brings over your bill. The bill, like the one shown in Fig. 1, will almost always include three types of number: cardinals, ordinals, and nominals (Nieder, 2005; Wiese, 2004). Cardinal numbers on the bill count sets (e.g., two portions of white rice, seven total items) and quantify elements of conventional scales (e.g., the prices of each item, such as £1.00 for a cup of Kurdish tea), answering the questions “how many?” or “how much?”. Ordinal numbers on the bill, such as the date (e.g., “05/06/2023”) and time (e.g., “18:55:09”), index a position in an ordered sequence (e.g., months in a year, minutes in an hour). In comparison with cardinals, ordinals do not involve quantification. Lastly, nominal numbers on the bill, such as the restaurant's telephone number (e.g., “01509767667”) and postcode (e.g., “LE11 3DU”), act as unique identifiers, “like proper names” (Wiese, 2004, p. 11). The only necessary property of these nominals is that they are unique in a given context, so their selection does not have to involve either quantification or ordering.^1^

Several studies have explored the frequency with which morphologically unmarked numbers (e.g., “one” and “30,” but not “first” and “30th”) are used (Coupland, 2011; Dehaene & Mehler, 1992; Dorogovtsev, Mendes, & Oliveira, 2005; Jansen & Pollmann, 2001; Woodin, Winter, Littlemore, Perlman, & Grieve, 2024), showing, for example, that people use smaller numbers more frequently than larger ones and round numbers more frequently than unround ones. These studies tend to assume, either explicitly or implicitly, that cardinal numbers are the most prototypical number type (Wiese, 2004, p. 2), and thus that the analyzed numbers reflect mostly quantitative uses (see discussion in Cummins, 2015, Ch. 6). This assumption is problematic when aggregated number frequencies are cited to make claims about, for example, the linear or logarithmic scaling of numerical cognition (Dehaene & Mehler, 1992) or the imprecision of the approximate number system (Woodin et al., 2024; see also Rinaldi & Marelli, 2020). These claims are predicated on quantitative uses of numbers, so the extent to which they are warranted depends on to what extent the number frequency data capture quantitative, cardinal uses. The research literature also contains untested claims about non‐cardinal numbers, some of which conflict. For example, Nieder (2005, p. 178) describes nominals as “atypical,” whereas Wiese (2004, p. 11) makes the claim that “nominal assignments are actually quite common in our daily lives.” Both statements relate to the frequency with which nominals are used, relative to cardinals and ordinals, but no study has assessed which type of number, if any, is more or less common in contemporary, industrialized cultures. Information about the relative frequencies of number types is relevant to studies that have investigated the order in which these number types are acquired by children (e.g., Colomé & Noël, 2012; Fischer & Beckey, 1990; Miller, Major, Shu, & Zhang, 2000) because input frequency could explain why certain number types are acquired earlier or later than others.

This paper is the first to conduct a corpus analysis of number types, exploring the relative proportions of cardinal, ordinal, and nominal numbers in a corpus of American English. In doing so, we establish a necessary initial baseline for analyses of number frequencies whose hypotheses implicitly relate to cardinal uses of numbers, particularly those based on large‐scale natural language data. Overall, this paper provides a first snapshot into how numbers are used in context, shedding new light on numerical communication, as well as numerical and mathematical cognition.

## 2 Background

Several studies have investigated frequencies for morphologically unmarked numbers in corpora. Dehaene and Mehler (1992) looked at single number words (i.e., no multiword phrases, e.g., “one hundred and twenty one”) from 0 to a billion in an American English corpus of more than 1 million words (Francis & Kučera, 1982). They found that number frequency decreases with magnitude in a logarithmic fashion, citing this trend to support the idea that numerical cognition itself is logarithmically scaled. They also found that the round numbers 10, 20, 50, and 100 are used frequently, relative to their magnitude. Other studies have reported similar patterns regarding magnitude and roundness for numbers 2–1,000 in approximation contexts (preceded by “about”) in a 40 million word corpus of articles from the English newspaper *The Times* from 1994 (Jansen & Pollmann, 2001); numbers 1–20 in the 100 million word British National Corpus (BNC Consortium, 2007) and numerals and English number words 1–100 on the Internet (both: Coupland, 2011); the frequencies of web pages containing numerals (Dorogovtsev et al., 2005); and numbers 0 to a billion in the British National Corpus (Woodin et al., 2024). However, none of these studies distinguished between cardinal, ordinal, and nominal numbers.

There is no easy way of differentiating between different uses of numbers without investigating each number closely in its communicative context. Dehaene and Mehler (1992) examined number words such as English “second” and “nineteenth,” whose morphology suggests that they are ordinal. However, many English ordinals are not morphologically marked, such as when people talk about their “top 5 songs.” Thus, the same number, sometimes without morphological adjustment, can have an ordinal reading when embedded in a construction such as “top *N*” or “bottom *N*,” but a cardinal reading when embedded in a construction such as “5 people.” Even morphologically marked words like “fifth” and “eighth” that may usually be ordinal can have a cardinal meaning in some contexts, as in fractions (e.g., “an eighth of a cup of butter”). Other English constructions cue a nominal interpretation, like when a number is preceded by the word “number” itself, as in “bus line number 23” and “player number 23” (Wiese, 2004, pp. 270–274). However, it is also possible to cue a nominal reading *without* the word “number,” as in “bus line 23” and “player 23,” and there are nominal uses of numbers that do not follow this pattern at all, such as “call 911.” Therefore, the task of identifying different number types is more complicated than simply identifying numbers with certain morphologies or in pre‐defined constructions, requiring a careful reading of the contexts in which these numbers are used.

Using corpus methods to identify number types and report their relative frequencies is important for the study of many topics relevant to numerical and mathematical cognition. One of these topics is children's acquisition of number concepts. While most research in this area has explored the acquisition of cardinal numbers, some studies have compared the relative order with which ordinal and cardinal numbers are acquired by child speakers of Belgian French (Colomé & Noël, 2012), U.S. English (Fischer & Beckey, 1990), and U.S. English and Mandarin Chinese (Miller et al., 2000), finding that cardinal uses of numbers are acquired before ordinals. There are no data on the acquisition of nominals relative to cardinals and ordinals, but it is presumed that they are acquired later (see discussion in Wiese, 2004). However, studies that look at the acquisition of different number types by children would be informed by the relative uses in adults in their respective languages, if we take adult language to be the target of acquisition. For example, nominals may be acquired later because they are less frequent in the language input children receive, rather than this late acquisition having anything to do with nominals being more cognitively difficult. Thus, corpus analyses of the relative frequency of cardinals, ordinals, and nominals would establish baselines that could inform developmental research in mathematical cognition.

Such corpus analyses of number types are also relevant to debates about whether people think about numbers linearly or logarithmically (e.g., Dehaene, 2003; Moeller, Pixner, Kauffman, & Nuerk, 2009; Piantadosi, 2016). As well as behavioral research, language data are often cited as a source of evidence in these debates: Dehaene and Mehler (1992) argue that the higher relative frequency of smaller numbers in corpora of English and other languages (e.g., Dutch, Japanese, Kannada) supports a logarithmic model. However, claims about the scaling of numerical cognition concern quantification, and so are predicated on cardinality, when cardinal, ordinal, and nominal numbers are conflated in the data cited to corroborate these claims. Thus, it is possible that number type is a confounding factor in these analyses, as the relationship between frequency and magnitude is likely to differ between number types. For example, ordinals and nominals may tend to be smaller than cardinals because, among other reasons, reports of ordinal rank usually mention the very best or worst cases of a category (e.g., “top 10 university”), and nominal player numbering usually starts at 1, and sports teams usually do not have hundreds or thousands of players. In comparison, quantitative uses of numbers can assume far higher values, such as money (e.g., “a billion dollars”). If ordinals and nominals do skew smaller than cardinals, they would drive up frequencies of smaller numbers in corpus studies investigating morphologically unmarked numbers, potentially presenting an issue when arguments about the logarithmic scaling of the mental number line are predicated on these studies.

Relative frequencies of number types are also relevant for new methods of studying the approximate number system in distributional semantics, a computational approach that represents meanings as word vectors based on the contexts in which words are used. For example, even when “doctor” and “physician” do not appear in the same texts, they tend to appear in the same kinds of text (Günther, Rinaldi, & Marelli, 2019), providing a cue to their meaning, following Firth's (1957, p. 179) famous credo that “you shall know a word by the company it keeps.” Using distributional semantics, Rinaldi and Marelli (2020) demonstrated they could derive several properties associated with the approximate number system from text data alone for English and other languages (e.g., German, Russian, Chinese). Taking vector variance as a proxy, they showed that larger numbers are used less precisely than smaller numbers, just as the approximate number system is argued to be increasingly imprecise for larger quantities (DeWind, Adams, Platt, & Brannon, 2015; Shepard, Kilpatric, & Cunningham, 1975). However, these results are predicated on word vectors derived from *all* contexts in which morphologically unmarked numbers are used, while the approximate number system is only relevant to quantitative, cardinal contexts. Just as is the case with the other studies mentioned above, it is possible that Rinaldi and Marelli's (2020) results are affected by the distribution of cardinals, ordinals, and nominals changing as a function of magnitude. For instance, if small numbers are relatively more likely to be ordinal or nominal (as suggested above), vector variance may increase for larger numbers because larger numbers are more likely to be cardinal, and cardinals may be used less precisely *in general*. If true, Rinaldi and Marelli's (2020) word vectors would not necessarily reflect the approximate number system.

Consistent with the idea that numerical communication is less precise for larger numbers, Woodin et al. (2024) showed that at higher magnitudes, users of British English tend to use more round (morphologically unmarked) numbers, defined as multiples of 10 and sometimes 5 in the decimal system typically used in English (e.g., Dorogovtsev et al., 2005; Sigurd, 1988). Round numbers are often (but not always) used imprecisely (e.g., Jansen & Pollmann, 2001; Krifka, 2009; Sigurd, 1988), such as when rounding 248 up to 250 when counting attendees at a conference. However, Woodin et al. (2024) also did not differentiate quantitative, cardinal uses of numbers from nominal and ordinal uses, and it is unclear to what extent roundness governs the use of nominals and ordinals, given that quantification is the prototypical context for rounding (e.g., “around 100 cars,” “nearly 20 meters high”). Roundness is probably irrelevant to nominals, as rounding nominals alters their referent: for example, rounding Cristiano Ronaldo's player number 7 to 10 would denote another player entirely. In comparison, one study showed that French speakers do round ordinals in time‐telling contexts, such as rounding 3:08 to 3:10 (Van der Henst, Carles, & Sperber, 2002; see also Gibbs & Bryant, 2008), and United Kingdom university reports often structure “top N” lists around multiples of 5, as in “Warwick repeats top 10 success” (Cummins & Franke, 2021, p. 12). Furthermore, when interpreting ranked lists, people more favorably interpret rank improvements that cross round number boundaries (e.g., rank 11 to rank 10) than those that do not (e.g., rank 10 to rank 9) (Isaac & Schindler, 2014). Therefore, roundness may influence the frequency with which round and unround ordinals are used to some extent, although it is unclear whether this factor is as relevant to ordinals as it is to cardinals.

Relating the use of round numbers to the approximate number system is further complicated by the idea that the kinds of number considered appropriate for use in approximation contexts in English may differ between subtypes of cardinal number: “pure” cardinal numbers that count sets (people, plants, buildings, etc.) and measurements that quantify elements of conventional scales (cost, length, duration, etc.). Particularly, approximate use of measurements, unlike pure cardinals, may not exclusively involve round numbers: Cummins (2015, Ch. 6) argues that whereas the English expression “about 19 people” is infelicitous, “about 19 minutes” is felicitous, because it implies that “19 minutes” has been rounded to the nearest minute (e.g., from 18 minutes 47 seconds). This difference derives from measurement scales being recursively divisible into subscales, like seconds within minutes, milliseconds within seconds, and so on. Rounding measurements in English may therefore involve rounding subscales to the nearest scale unit, rather than necessarily to multiples of 5 and 10. Consequently, the trend for multiples of 5 and 10 to be used more frequently at higher magnitudes in English may be stronger for pure cardinals, as measurements may rely less on multiples of 5 and 10 for approximation. If so, another approach that does not investigate imprecision exclusively through the use of conventionally “round” numbers may be required to obtain an accurate picture of approximation across magnitude.

The use of cardinal, ordinal, and nominal numbers may also differ across registers: varieties of the same language that differ in terms of their communicative context or goal (Trudgill, 1983, p. 101). A major distinction here is based on whether a register is spoken or written: Biber (2012) cites a range of research showing that language use varies drastically between speech and writing in English, particularly academic writing (e.g., Biber & Gray, 2010; Conrad & Biber, 2009; Kennedy, 1998). Other work has found differences between more specific registers, for example, between manuals, letters, and novels in English and German (Neumann, 2014), and narration and speech within English novels (Egbert & Mahlberg, 2020). Regarding numerical language, Woodin et al. (2024) found that registers in British English speech and writing, respectively, such as non‐fiction writing and oral presentations, contained more decimals and larger numbers, and more unique numbers, than less informational registers. As discussed above, however, this study did not distinguish between cardinals, ordinals, or nominals. Other studies have exclusively investigated cardinal numbers, finding that English news texts are quantitatively dense (e.g., Cushion, Lewis, & Callaghan, 2017; Maier, 2002). It is possible that news texts use numbers for their purported ability to boost perceived objectivity (Barchas‐Lichtenstein et al., 2022; Porter, 1995) and credibility (Koetsenruijter, 2011). Exploring the distribution of number types across registers (e.g., academic writing, fiction writing) can tell us what types of numerical information (e.g., quantitative, non‐quantitative) are relevant in different communicative contexts with different goals.

In writing, different number types may be represented in different representational formats—numerals (e.g., “122”) and number words (e.g., “one hundred and twenty‐two”; see Chrisomalis, 2020, Ch. 6). Whether a number is written as a numeral or as a number word is often prescribed by style guides (e.g., The Chicago Manual of Style, 2017b). For example, nominal phone numbers and zip codes, as well as ordinal years and page numbers, are probably almost always represented as numerals in English; presumably, nobody would write the U.S. zip code 95340 as “nine five three four zero,” for example. In contrast, cardinals do not prescribe representational format as strongly; for instance, both “30 people” and “thirty people” are felicitous. Within cardinals, it is also possible that measurements are more likely to be written as numerals than pure cardinals for reasons of concision: in English, units of measurement are generally allowed to be abbreviated, or represented as a symbol when paired with numerals (e.g., “12 mm,” “$100”) but not number words (e.g., “twelve millimeters,” “one hundred dollars”). In cases where representational format is more flexible (e.g., for cardinals), there may be greater pressure to keep unround numbers short by representing them as numerals, given that unround number word expressions are typically longer than round ones (e.g., “two hundred and one” vs. “two hundred”). Moreover, certain representational formats may be preferred for certain number types: for example, mixtures of numerals and multiplier words (e.g., “$13 trillion,” “2.5 billion”) are exclusively selected for cardinal meanings in English. Therefore, representational format itself can serve as a cue that helps readers to infer how numbers are used—a probabilistic cue, rather than the more categorical cue provided by constructions (e.g., “bus line number 23,” “player number 23”; Wiese, 2004, pp. 270–274) and morphology (e.g., “sixth,” “10th”).

In this paper, we present the first corpus analysis investigating the different types of numbers used in modern, industrialized societies: cardinals (pure cardinals, measurements), ordinals, and nominals. We focus on morphologically unmarked numbers (e.g., “3” and “20,” not “3rd” and “twentieth”), as these are the types of numbers typically aggregated in analyses of number frequencies that are often cited to make claims about cognition. These numbers also seem to be more ambiguous when divorced from context than morphologically marked numbers, which are likely to be predominantly ordinal, with, as mentioned above, the exception of their use in fractions. We conduct this analysis by manually annotating a sample of concordances from the Corpus of Contemporary American English (COCA; Davies, 2008). Concordances, or concordance lines, are lines of text that show how words (in our case, numbers) are used in context. With these concordances, we investigate the relative frequencies of the number types overall and in relation to factors such as magnitude, roundness, register, and, in writing, representational format, in addition to the interaction between these factors. In doing so, we delve deep into the connection between numerical communication and important aspects of numerical cognition and mathematical cognition.

## 3 Methodology

All data, analysis scripts, and detailed information about the methodology described in this section can be found in the following OSF repository: https://osf.io/mdtb4/.

### 3.1 Corpus

We used the COCA (Davies, 2008), a large reference corpus created to be representative of spoken and written American English by sampling a range of texts and registers, intending to allow generalizations across different types of language use. COCA was released in 2008 and has been updated continuously on an annual basis since then. The 2014 version to which we had access comprises 115 texts containing 440 million words, and the following subcorpora: speech, fiction, magazines, newspapers, and academic journals. The speech subcorpus comprises casual conversations from TV and radio programs (e.g., *Newshour, Jerry Springer, Good Morning America*). The fiction subcorpus comprises short stories and plays published in magazines, books published from 1990 onward, and film scripts. The magazine subcorpus comprises magazines roughly balanced for topic, such as finance, home, and religion (e.g., *Time, Cosmopolitan, Men's Health*). The newspaper subcorpus comprises a mix of sections (e.g., local news, opinion, sport) from U.S. newspapers (e.g., *New York Times, USA Today, San Francisco Chronicle*). The academic journal subcorpus contains peer‐reviewed articles with texts from different subject areas (e.g., philosophy, psychology, world history).

As a caveat, although COCA is intended to be broadly representative of American English—and is successful in this goal in many ways for the reasons described above—it notably only covers spoken language on TV and radio, and no informal or private registers, such as writing on social media or speech in peer‐group conversations. Moreover, all subcorpora arguably except fiction writing contain at least some informational elements (see Woodin et al., 2024), including the speech subcorpus, which includes conversations on news programs. Therefore, the corpus may be biased toward contexts in which factual information is being conveyed. COCA also features texts from a limited time frame (1990 onwards), which is likely to influence number frequencies with respect to reporting of years. These limitations should be borne in mind when interpreting the findings presented in this paper.

### 3.2 Software

To extract concordances containing numbers from COCA, we used Python (version 3.7; Python Software Foundation, 2021) inside the integrated development environment PyCharm (version 2022.2.3; JetBrains, 2022) with the built‐in libraries Re (version 2.2.1), Time, OS, and IterTools (all: Van Rossum, 2020), and the external libraries Pandas (version 1.1.5; McKinney, 2010), NLTK (version 3.7; Bird, Klein, & Loper, 2009), Word2Number (version 1.1; Batorsky, Ledvosky, Yarkoni, & Groove, 2021), and Num2Words (version 0.5.12; Dupras, Ortiz, Szalaï, Lodato, & Harper, 2021). To perform the main analyses of the annotated concordances, we used programming language R (version 4.1.1; R Core Team, 2021) inside the integrated development environment RStudio (version 2023.6.0.421; Posit Team, 2023) with the packages “tidyverse” (version 1.3.1; Wickham et al., 2019) and “ggpubr” (version 0.4.0; Kassambara, 2020).

### 3.3 Extracting concordances

The procedure used for extracting concordances containing numbers was adapted from Woodin et al. (2024). We aimed to extract concordances that contained numbers from 0 to 1 billion that were either transcribed (speech) or written (writing) as numerals (e.g., “12”, “1.5”), number words (e.g., “twelve,” “one point five”), or a mixture of numerals and number words (e.g., “1 billion,” “7.5 million”), where these numbers were morphologically unmarked (not, e.g., “2nd,” “second”). Each of these concordances was 150 characters long.

To extract the concordances, we used the Python library NLTK to parse COCA, part‐of‐speech tag each string (numerals or number words), and extract all strings that were tagged as numbers. NLTK tags each string individually, so to identify numerical expressions comprising multiple words (e.g., “one hundred and one,” “two point five”), we captured as a whole all adjacent numerical strings, also capturing the words “and” and “point.” Our use of part‐of‐speech tagging allowed us to extract numbers in a bottom‐up manner and thereby capture a much greater range of numerical language than other studies that searched for specific numbers in a top‐down manner (Coupland, 2011; Dehaene & Mehler, 1992; Dorogovtsev et al., 2005; Jansen & Pollmann, 2001). The latter approach can only make inferences about numbers included in the search, rather than all numbers occurring in a corpus, as is the case in the present paper. Moreover, our approach was able to distinguish numerical uses of “one” from pronominal uses (e.g., “as one does”) (cf. Cerri, 2020, p. 67). However, this approach also necessitated data exclusions to remove concordances with numbers that did not meet the criteria described above for morphologically unmarked numbers. For instance, we excluded 627,596 strings containing a mix of both numerals and letters or other characters, including ordinals (e.g., “from 10th to 11th magnitude”), year ranges (e.g., “1980s”), and Bible book and verse numbers (e.g., “Luke 2:11”). We also excluded 1,667 number words like “eleven fifty nine” that were ambiguous between, for example, prices (e.g., “£11.59”), times (e.g., “11:59”), and years (e.g., “1159”). For more information about this procedure and all data exclusions, see the OSF repository associated with this paper (https://osf.io/mdtb4/) and the detailed description in Woodin et al. (2024).

Overall, this procedure identified 8,158,113 concordances containing numbers that met the above criteria. In a separate analysis not reported in our main results, we also extracted numerals and number words from 0 to a billion that were morphologically marked (e.g., “6th,” “sixth”). This extraction procedure identified 1,259,237 numbers, which comprises 13.4% of the combined total of 9,417,350 unmarked and marked numbers. These morphologically marked numbers—most of which we may assume are ordinals—then, are relatively frequent, although much less frequent than the unmarked numbers. We comment on these morphologically marked numbers in the discussion.

The rest of the paper focuses on the 8,158,113 unmarked numbers, which is the superset from which we sampled a subset of concordances for manual annotation, allowing us to analyze how each number was used in context.

### 3.4 Sampling concordances

We took a sample of 3,600 concordances, stratified across four criteria. First, we sampled concordances containing numbers from across the entire 0 to 1 billion range, as we are interested in whether the frequencies of number types differ with magnitude. Second, we stratified our sample across spoken and written American English because, as well as this being a prominent distinction in COCA, previous research attests to a wide range of linguistic differences between spoken and written English (e.g., Biber & Gray, 2010; Conrad & Biber, 2009; Kennedy, 1998). Third, in the written subcorpora (fiction, newspapers, magazines, academic), we stratified our sample by representational format—numerals and number words—as we are interested in whether there are differences in representational format for the different number types across magnitude. We did this only for writing, where representational format is a decision made by a text's author, and not speech, where representational format reflects the number's transcription and not how the number was actually spoken (see Woodin et al., 2024). Fourth and finally, we stratified our sample across round and unround numbers, as we are interested in whether roundness governs the frequency of certain number types to the same degree across magnitude, and if roundness influences the written representational format used for certain number types.

Table 1 shows the number of concordances sampled as a function of order of magnitude (rows), subcorpus (columns), and representational format (subcolumns). The 3,600 concordances we sampled comprised 200 concordances each for the nine orders of magnitude [1–10], (10–100], (100–1,000], (1,000–10,000], (10,000–100,000], (100,000–1 million], (1 million–10 million], (10 million–100 million], and (100 million–1 billion] in speech and separately for numerals and number words in writing (100 concordance lines each per order of magnitude). We did not include numbers written as a mixture of numerals and multiplier words (e.g., “1 million,” “2.5 billion”) in the written subsamples, as these invariably have a cardinal meaning in English.^2^ We ensured that at least 25% of each subsample comprised round numbers and unround numbers, respectively, providing there were enough of each type available to sample. We sampled the remaining 50% (or more) of each subsample at random from the remaining concordances. We operationalized numbers as round if they were multiples of 5 because Woodin et al. (2024) found this property to be the minimum requirement for numbers to be considered round in English. By this definition of rounding, the sample overall comprised 2,211 round numbers (61.4%) and 1,389 unround numbers (38.6%).

**Table 1:** Number of concordance lines analyzed per order of magnitude (rows), subcorpus (columns), and representational format (subcolumns)

		Written COCA	
Order of Magnitude	Spoken COCA	Numerals	Number Words
[1–10]	200	100	100
(10–100]	200	100	100
(100–1,000]	200	100	100
(1,000–10,000]	200	100	100
(10,000–100,000]	200	100	100
(100,000–1 million]	200	100	100
(1 million–10 million]	200	100	100
(10 million–100 million]	200	100	100
(100 million–1 billion]	200	100	100

*Note*. Consistent with mathematical convention, [] is used for orders of magnitude that include the numbers listed, whereas (] is used for orders of magnitude excluding the lower numerical limit. At least 25% of each subsample comprised round numbers and unround numbers, respectively, if there were enough round and unround numbers to sample.

It should be noted that, in a similar vein to Jansen and Pollman (2001), Woodin et al. (2024) found roundness to be a matter of degree rather than being categorical, with certain numbers being “rounder” than others (e.g., 1,000 > 7,500 > 55,005). Thus, nuance is necessarily lost in our more simplistic definition based on divisibility by 5. However, all the numbers considered round in Woodin et al. (2024) were multiples of 5, and divisibility by 5 was found to be a statistically reliable predictor of number frequency independently of other factors (divisibility by 10 and Jansen and Pollman's, 2001, roundness properties). As a consequence, even if a number like 55,005 is not the most prototypical example of a round number, it may nonetheless be “rounder” than a number like 55,003. In any case, as we will demonstrate, trends in the use of round numbers are evident using our operational definition, and due to this definition's simplicity may be clearer than if more nuanced, complex criteria were applied. Moreover, while nominal numbers are not “round” in the traditional sense—it may seem odd to describe a telephone number ending in 5 or 0 as “round” per se—we still see “roundness” as a useful shorthand that facilitates comparison with the other number types. Similarly, strictly speaking, nominals do not have a magnitude—a telephone number like “911,” for example, is a unique series of digits that would typically be spoken aloud as “nine one one” rather than “nine hundred and eleven”—but grouping the nominals into different orders of magnitude facilitates insightful comparisons between nominals and the other two number types.

### 3.5 Annotation of concordances

We developed a manual for annotating concordances (see https://osf.io/mdtb4/) that was iteratively updated in response to seeing the data. This more exploratory approach was needed as we did not know prior to seeing the data what patterns we would find. Such an iterative, bottom‐up approach is common in corpus linguistics. The annotation of concordances comprised two main stages.

First, we coded whether numbers were pure cardinals (e.g., “out of a million letters,” “upwards of 300,000 deaths”), measurements (e.g., “$50 million,” “four hundred pounds of cotton”), ordinals (e.g., “1977 Community Reinvestment Act,” “favorite Top 1,000 songs”), or nominals (e.g., “911 call,” “Tucson 85721”). Some concordances were coded as having a nominal meaning that was derived from ordinal information (e.g., “635 Massachusetts Avenue,” “Bates no. 480030740”) (see Wiese, 2004) or cardinal information (e.g., “the S&P 500” = stock market index name based on the number of companies it tracks, “SAO 78349” = star name derived from its interstellar position). Other concordances referred to numbers themselves in an abstract, “meta” way (e.g., “the sequence 601324897,” “de answer is 2446”). With these criteria, we were able to analyze 3,503 concordances (97.3%). For the remaining 97 concordances (2.7%), we were unable to determine the number type (e.g., “OB 89,” “Smith. 20110526”), even after a careful reading of the concordance, and conducting an Internet search for additional information.

Second, to look in more detail at how pure cardinals, measurements, ordinals, and nominals are used in context, we annotated for what functions they are used, by which we mean the more specific information these numbers are used to communicate. For pure cardinals, these functions included counting people (e.g., “sample size of 14,” “thirty‐seven million Americans”) and objects (e.g., “160,000 vehicles a day,” “a million letters”)—not including buildings, which had their own category. For measurements, these functions included quantifying money (e.g., “$1,728,096,” “a billion rubles”), time (e.g., “10,000 years ago,” “past 10 days”), and distances and spatial measurements (e.g., “130,286 square feet,” “150 yards”). For ordinals, these functions included indexing years (e.g., “in 1995,” “1930 adobe residence”), days (e.g., “September 11,” “March 4”), and pages in texts (e.g., “Baisre and Cruz 1994, 122,” “recipe page 136”). For nominals, these functions included their use in communications numbers (e.g., “Fax; 34 932147302,” “650 347‐7231”), programming code used on websites (e.g., “15421101 ‘Video,” “* * 28; 16939”), and zip codes (e.g., “Urbana IL 61801,” “Emmaus PA 18098”). For each number type, we included a miscellaneous category for functions that did not fit into the main categories identified (e.g., for cardinals: “hundred data sets,” “the thousand points of light”).

### 3.6 Exclusions

We excluded the following concordances from all analyses:

- Three hundred seventy‐four concordances (10.4% of full dataset) that had a meta‐function within the COCA texts, marking the order of different text sections, which were not part of the texts themselves but rather were added later as part of the organization of the corpus (e.g., “## 4072972,” “# # 231149”).
- Thirty‐seven concordances (1.0% of full dataset) containing cardinal numbers that were ambiguous between pure cardinal and measurement uses, because the context provided by the concordance did not provide enough information to determine how a number was used (e.g., “fifteen million per year,” “between thirty thousand and forty thousand more are needed”).
- Four concordances (0.1% of full dataset) that were concatenations of different number types. For example, an instance of the number “30199998” was a concatenation of 30 (August 30th), 1999 (the year), and 98 (a percentage). These concatenations were an unanticipated outcome of our number identification procedure, which involved removing punctuation from texts. Concordances that were concatenations of multiple numbers of the same type and function (e.g., “19861987” = ordinal years “1986” and “1987”) were retained for analyses where the precise magnitude of the number did not matter (Sections 4.1 and 4.2) but were only counted once in these analyses (e.g., we did not count “1986” and “1987” separately).
- Four concordances (0.1% of full dataset) containing non‐numerical uses of numbers, including three pronominal uses of “one” that were accidentally captured by NLTK (e.g., “one would have to think”), and one number homophone (“all the way 2 pluto and bac,” where “2” means “to”).

After these exclusions, the final dataset comprised 3,181 concordances.

## 4 Results

This section begins with an overview of how many concordances are cardinal (either pure cardinal or measurement), ordinal, nominal, or otherwise, and the main functions of these numbers (Section 4.1). We then report the frequency of each number type across registers (Section 4.2) and magnitudes (Section 4.3), and the extent to which number types of different magnitudes are round or unround (Section 4.4). Finally, in writing (fiction, newspapers, magazines, academic), we report the extent to which the different number types are written as number words and numerals, and whether this representational format depends on roundness (Section 4.5).

### 4.1 Overall

**Fig. 2:**
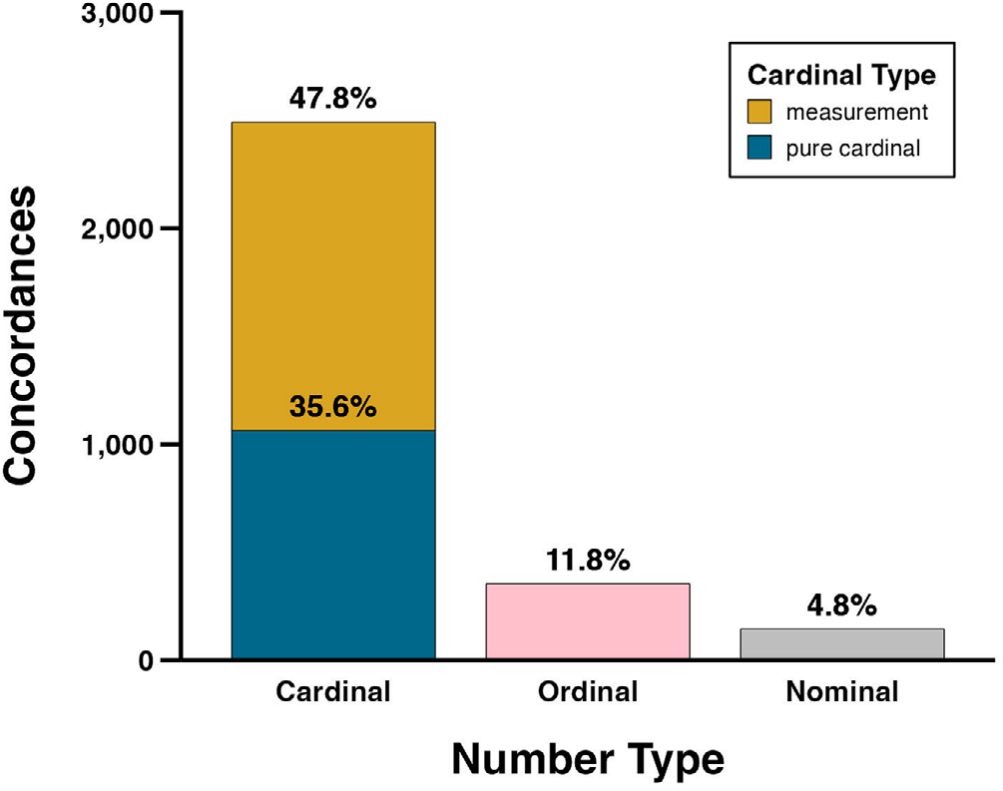
Counts (bars) and percentages (text) of concordances containing cardinal, ordinal, and nominal numbers, with cardinal numbers subdivided into pure cardinals and measurements.

Fig. 2 displays the proportion of cardinal, ordinal, and nominal number types overall. Cardinal numbers are subclassified as pure cardinals or measurements. This figure shows that cardinals are far more frequent (83.3%, *N* = 2,491) than the ordinals (11.8%, *N* = 354) and nominals (4.8%, *N* = 144). Within the cardinals, measurements (47.8%, *N* = 1,428) are more frequent than pure cardinals (35.6%, *N* = 1,063).^3^

Table 2 displays the top 10 most common functions for which pure cardinals, measurements, ordinals, and nominals are used (there are only seven functions for the nominals). Full lists of each function and explanations can be viewed at https://osf.io/mdtb4/. For pure cardinals, the most common function is counting people (51.5%, *N* = 547), followed by counting objects (11.7%, *N* = 124), and the “Other” category (9.2%, *N* = 98), in which numbers quantify things like “canals,” “ideas,” and “public health decisions.” For measurements, the most common function is quantifying money (54.4%, *N* = 777), followed by time (18.6%, *N* = 266), and distances and spatial measurements (8.6%, *N* = 123). For ordinals, the most common function—to a greater degree than the other number types—is indexing years (84.1%, *N* = 290), followed by days (3.8%, *N* = 13), and page orders (2.3%, *N* = 8) (excluding nine concordances comprising concatenated ordinals with different functions, e.g., “Jan 271998” = “27” day + “1998” year). Nominals most often occurred in communications numbers (31.2%, *N* = 45), programming code used on websites (31.2%, *N* = 45), and zip codes (24.3%, *N* = 35).

**Table 2:** Percentages and counts for the 10 most common functions for which pure cardinals, measurements, ordinals, and nominals are used (there are only seven functions for nominals)

Pure Cardinal			Measurement			Ordinal			Nominal		
Function	%	*N*	Function	%	*N*	Function	%	*N*	Function	%	*N*
People	51.5	547	Money	54.4	777	Year	84.1	290	Communications	31.2	45
Objects	11.7	124	Time	18.6	266	Day	3.8	13	Web codes	31.2	45
Other	9.2	98	Space	8.6	123	Page	2.3	8	Zip codes	24.3	35
Events/Actions	5.6	59	Age	4.7	67	Time	2.0	7	Other	5.6	8
Jobs	2.6	28	Percentage	4.6	65	Other	1.7	6	Legal number	3.5	5
Buildings	2.4	26	Mass	3.9	55	Section	1.4	5	Product name	2.1	3
Organizations	2.0	21	Other	1.8	25	Unknown	1.4	5	Road name	2.1	3
Unknown	1.9	20	Capacity	1.5	21	Citation	1.2	4			
Points	1.9	20	Concentration	0.8	12	Top N	1.2	4			
Occurrences	1.7	18	Temperature	0.7	10	Recipe	0.9	3			

*Note*. Full lists of functions and explanations of these functions can be viewed at https://osf.io/mdtb4/.

### 4.2 Register

**Fig. 3:**
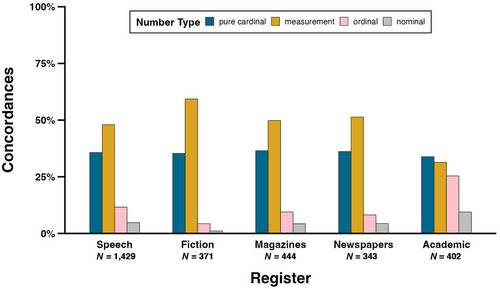
Percentages of concordances in each register of COCA that contain pure cardinals, measurements, ordinals, and nominals.

Fig. 3 displays the percentages of concordances containing each number type in each COCA register. For speech, fiction, magazines, and newspapers, the relative percentages mirror the overall results (see Section 4.1): measurements are most frequent (speech: 47.9%, *N* = 685; fiction: 59.3%, *N* = 220; magazines: 49.8%, *N* = 221; newspapers: 51.3%, *N* = 176), followed by pure cardinals (speech: 35.7%, N = 510; fiction: 35.3%, *N* = 131; magazines: 36.5%, *N* = 162; newspapers: 36.2%, *N* = 124), ordinals (speech: 11.6%, *N* = 166; fiction: 4.3%, *N* = 16; magazine: 9.5%, *N* = 42; newspaper: 8.2%, *N* = 28), and nominals (speech: 4.8%, *N* = 68; fiction: 1.1%, *N* = 4; magazine: 4.3%, *N* = 19; newspaper: 4.4%, *N* = 15). In contrast, in academic writing, pure cardinals are most frequent (33.8%, *N* = 136), owing to measurements being relatively less frequent (31.3%, *N* = 126). Compared to the other registers, ordinals are far more frequent (25.4%, *N* = 102), and nominals are also more frequent to a lesser extent (9.5%, *N* = 38). Ordinals in academic writing are likely to denote years (84.3%, *N* = 86), frequently found in citations, while nominals are often found in programming code used on websites (42.1%, *N* = 16), and zip codes (31.6%, *N* = 12), often in university addresses for academic authors.

### 4.3 Magnitude

**Fig. 4:**
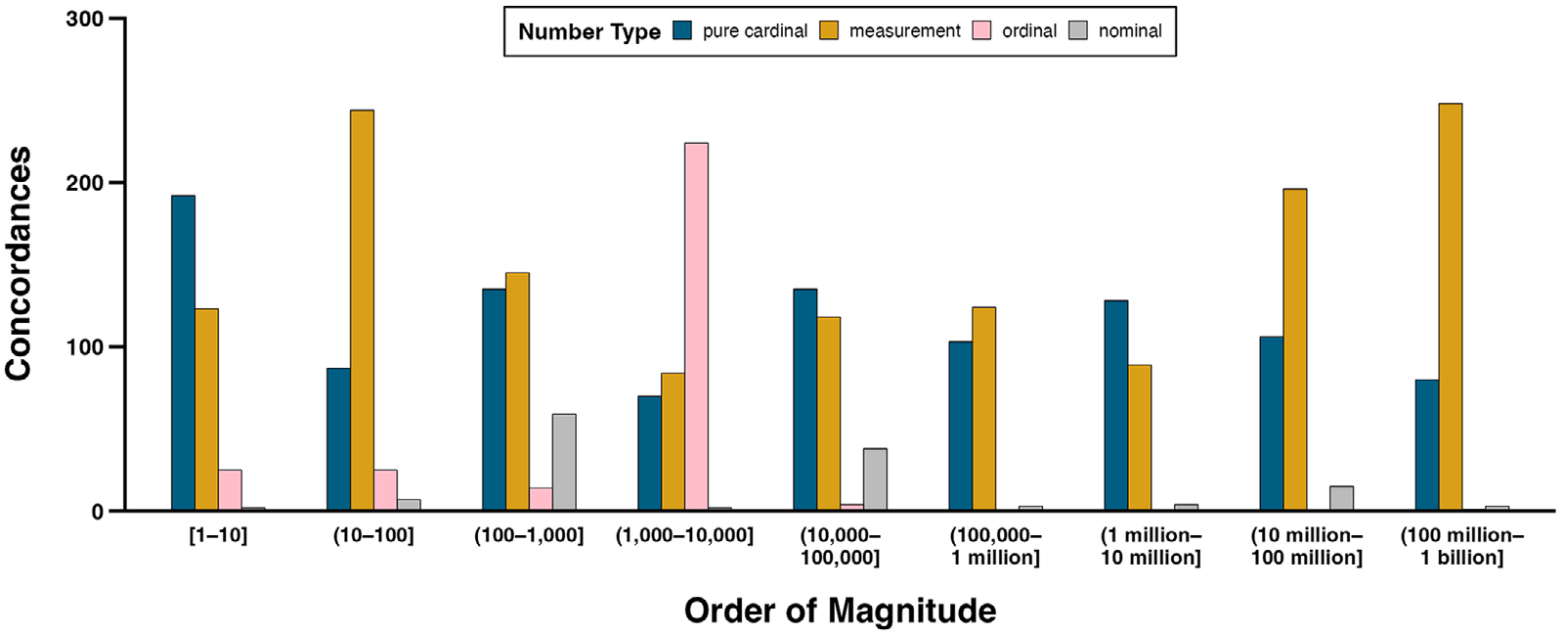
Counts of concordances containing pure cardinals, measurements, ordinals, and nominals of different orders of magnitude.

Fig. 4 shows the number of concordances that contain pure cardinals, measurements, ordinals, and nominals for nine orders of magnitude. Excluded from this and the following analyses are 159 concordances containing numbers whose precise magnitude was miscategorized (see Section 3.6 for details). When reporting results in‐text for different orders of magnitude in this and subsequent sections, round and square brackets are removed for legibility (e.g., “(1,000–10,000]” is written as “1,000–10,000”).

Fig. 4 shows that cardinals—both pure cardinals and measurements—dominate every order of magnitude except for 1,000–10,000. In relative terms, pure cardinals are dominant over measurements for numbers 1–10 (pure cardinals: 56.1%; *N* = 192; measurements: 36.0%, *N* = 123), while measurements dominate the numbers 10 million–1 billion (pure cardinals = 28.7%, *N* = 186; measurements: 68.4%, *N* = 444). The majority of these large measurements quantify monetary amounts (84.5%, *N* = 375, e.g., “twenty million dollar commitment”). Measurements also dominate the numbers 10–100 (measurements: 62.7%, *N* = 244; pure cardinals: 24.0%), with most of these measurements quantifying durations (40.2%, *N* = 98, e.g., “25 years to life”), followed by percentages (20.9%, *N* = 51, e.g., “25 percent or greater”), and ages (17.6%, *N* = 43, e.g., “at the age of 28”). Ordinals are the most frequent number type for numbers 1,000–10,000 (58.9%, *N* = 224), all of these ordinals indexing years (e.g., “the science of 1996”). Nominals are relatively infrequent regardless of magnitude but are somewhat more common for the numbers 100–1,000 (16.7%, *N* = 59). Most of these nominals are communications numbers (69.5%, *N* = 41, e.g., the area code “847” in “847 634–3291”).

### 4.4 Roundness

**Fig. 5:**
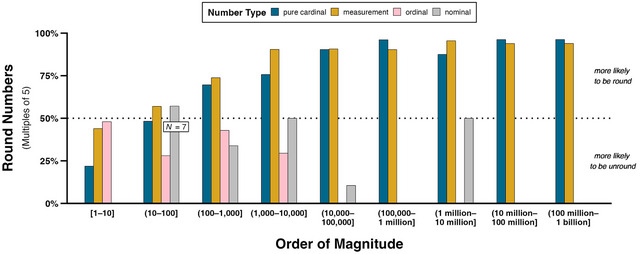
Percentages of concordances containing pure cardinals, measurements, ordinals, and nominals for each order of magnitude that are round (i.e., multiples of 5). The dotted line represents 50%; percentages above (under) this line thus indicate that numbers of a certain type and order of magnitude are proportionally more likely to be round (unround). Counts for nominals in order of magnitude (10–100] are superimposed on the figure to signal that, despite more than 50% of these nominals being round numbers, there are only seven concordances (four round numbers vs. three unround numbers).

Fig. 5 displays what percentage of each number type at different orders of magnitude is round, which, as discussed above, we operationalize as multiples of 5. Bars above the 50% line indicate that the majority of numbers are round. This figure reflects that, overall, round numbers are dominant for pure cardinals (round: 71.7%, *N* = 743; unround: 28.3%, *N* = 293) and measurements (round: 80.0%, *N* = 1,097; unround: 20.0%, *N* = 274), while unround numbers are dominant for ordinals (round: 31.1%, *N* = 91; unround: 68.9%, *N* = 202) and nominals (round: 23.3%, *N* = 31; unround: 76.7%, *N* = 102). For pure cardinals and measurements, the proportion of round numbers increases from 1 to 10,000 (pure cardinals: 43.0%, *N* = 178; measurements: 58.6%, *N* = 300) and remains high for the range 10,000–1 billion (pure cardinals: 92.8%, *N* = 512; measurements: 93.0%, *N* = 721). The only order of magnitude in which more pure cardinals are unround is 1–10 (78.1%, *N* = 150). Over half of these pure cardinals are the number 1 (58.0%, *N* = 87) and are frequently followed by “of” (39.1%, *N* = 34) in phrases like “one of the big advantages” and “one of my favorite rock legends.” More measurements of this order of magnitude are also unround, but to a lesser extent (56.1%, *N* = 69) compared to the pure cardinals. Finally, the relative proportions of cardinals in the other orders of magnitude are comparable, except for 1,000–10,000, where there is a higher proportion of unround pure cardinals (24.3%, *N* = 17) than unround measurements (9.5%, *N* = 8). Most of these unround pure cardinals are the number 1,001 (70.6%, N = 12), which appears in hyperbolic expressions like “a thousand and one extraordinary adventures” and “a thousand and one excuses.”

### 4.5 Writing: Representational format

#### 4.5.1 Overall

**Fig. 6:**
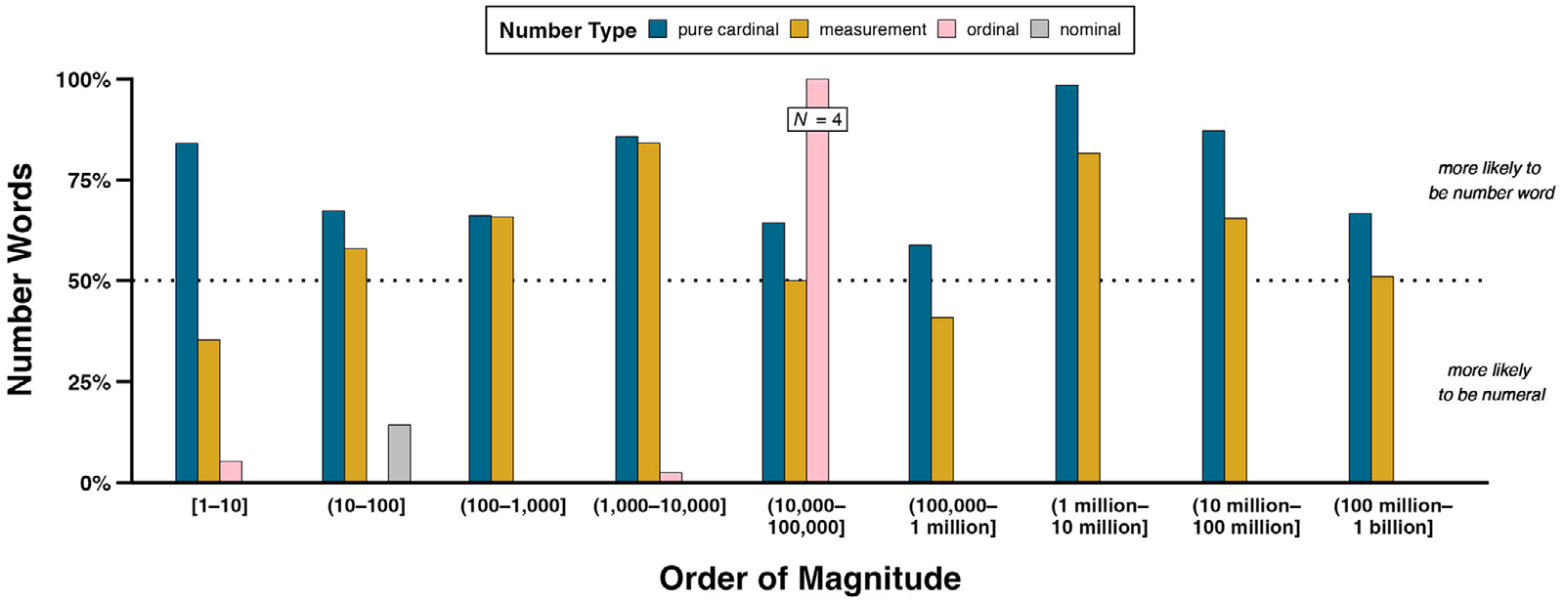
Percentages of pure cardinals, measurements, ordinals, and nominals for each order of magnitude that are represented as number words in writing. The dotted line represents 50%; percentages above (under) this line hence indicate that numbers of a certain type and order of magnitude are proportionally more likely to be number words (numerals). Counts for ordinals in order of magnitude (10,000–100,000] are superimposed on the figure to highlight that, despite all these ordinals being written as number words, there are only four concordances.

Fig. 6 shows the percentage of number types at different orders of magnitude that are represented as number words in writing. Thus, percentages over 50% indicate that number words predominate. This figure demonstrates that, across all magnitudes, pure cardinals are proportionally more likely to be number words (75.2%, *N* = 401) than numerals (24.8%, *N* = 132). In fact, all but one of the pure cardinals in the range 1 million–10 million are number words (number words: 98.5%, *N* = 66, numerals: 1.5%, *N* = 1), the most common of which is 1,000,001 (24.2%, *N* = 16), invariably used hyperbolically in expressions such as “a million and one questions” and “a million and one things.” Measurements overall are proportionally more likely to be number words (57.2%, *N* = 399) than numerals (42.8%, *N* = 298) as well, but to a lesser extent than pure cardinals: for no order of magnitude do measurements have a higher proportion of number words than pure cardinals. Overwhelmingly, ordinals (number words: 94.5%, *N* = 120; numerals 5.5%, *N* = 7) and nominals (number words: 1.5%, *N* = 1; numerals: 98.5%, *N* = 64) are written as numerals.

#### 4.5.2 Cardinals: Roundness

**Fig. 7:**
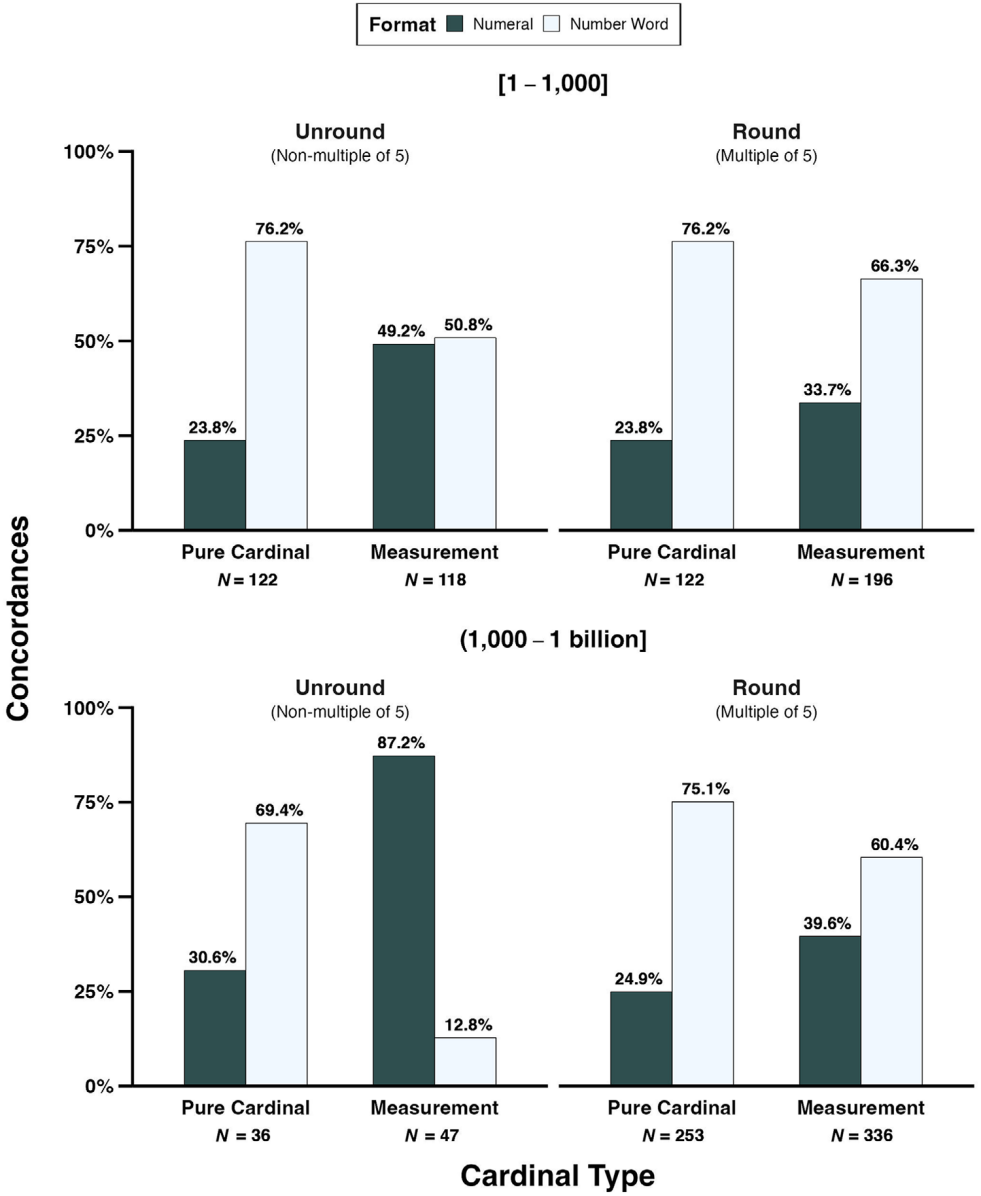
Percentages of concordances containing unround and round pure cardinals and measurements that are represented as numerals or number words in writing in ranges [1–1,000] and (1,000–1 billion].

Fig. 7 depicts the percentages of unround and round pure cardinals and measurements represented as numerals or number words for different numerical ranges: 1–1,000 (top row) and 1,000–1 billion (bottom row).^4^ We do not show percentages for ordinals and nominals as so few of these were represented as number words. This figure shows that, while pure cardinals are proportionally more likely to be written as number words across the board, unround measurements in the range 1–1,000 are about equally likely to be represented as number words or numerals (number words: 50.8%, *N* = 60; numerals: 49.2%, *N* = 58), and unround measurements in the range 1,000–1 billion are far more likely to be written as numerals (number words: 12.8%, *N* = 6; numerals: 87.2%, *N* = 41). Also, regardless of magnitude, the representation of round measurements as number words (number words: 62.6%, *N* = 333; numerals: 37.4%, *N* = 199) is notably lower than for the pure cardinals (number words: 75.5%, *N* = 283; numerals = 24.5%, *N* = 92).

## 5 Discussion

This study is the first corpus analysis investigating how often morphologically unmarked numbers are used as cardinals, ordinals, or nominals. Our results show that, in American English, cardinals—both pure cardinals and measurements—are dominant, followed by ordinals, then nominals. Cardinals dominate all orders of magnitude except 1,000–10,000, in which there is a high number of ordinal years, such as 1977 and 2024. Only for cardinals do round numbers (i.e., multiples of 5) dominate overall and increase with magnitude. Among quantitative uses of numbers, measurements are more common than pure cardinals, often quantifying money. In comparison with other registers, academic writing contains a lower proportion of measurements, as well as a higher proportion of ordinals and, to some extent, nominals. In writing, pure cardinals and measurements are usually represented as number words, but measurements—especially larger, unround ones—are more likely to be numerals. Ordinals and nominals are mostly written as numerals.

The relative frequencies of cardinals, ordinals, and nominals we report establish a baseline for analyses of aggregated number frequencies, whose hypotheses regarding numerical cognition hinge on quantitative, cardinal uses of numbers (Coupland, 2011; Dehaene & Mehler, 1992; Dorogovtsev et al., 2005; Jansen & Pollmann, 2001; Woodin et al., 2024), at least those analyses focused on English. Our findings generally confirm the assumptions of these previous analyses, showing that cardinals are the most prototypical number type (Wiese, 2004, p. 2) when it comes to morphologically unmarked numbers (i.e., not “twelfth,” “20th,” etc.), comprising 83.4% of concordances. As for nominals, our results support Nieder's (2005, p. 178) assertion that nominals are “atypical,” given that these are indeed the least often occurring number type. Nonetheless, nominals are still relatively “common” (Wiese, 2004, p. 11), comprising a non‐negligible 4.8% of concordances, which increases to 7.3% if we count the 81 concordances whose nominal meaning is based on ordinal (e.g., house numbers) or cardinal (e.g., “the S&P 500”) information. Ordinals are more frequent than nominals, comprising 11.8% of concordances. Therefore, we recommend that researchers at least acknowledge ordinals and nominals when aggregating number frequencies, even when cardinals are the focal point of the analyses.

In a separate procedure in addition to our main analysis reported above, we also identified 1,259,237 numbers whose morphology suggests they are ordinal (e.g., “tenth,” “13th”). These morphologically marked numbers represent 13.4% of the total combined unmarked and marked numbers. With this information, we can estimate the percentages of cardinals, ordinals, and nominals across all numbers in COCA (i.e., not just the morphologically unmarked ones, on which our main analysis is concentrated). We use the percentage of cardinals, ordinals, and nominals from our sample to estimate counts for all numbers in COCA. We then add to the ordinal count the 1,259,237 marked numbers, which we assume are ordinal, and estimate the percentages again. When we do this, 72.2% (*N* = 6,798,882) of all numbers are cardinal, whereas 23.6% (*N* = 2,225,437) are ordinal, and 4.6% (*N* = 393,031) are nominal.^5^ These statistics show that, compared to the main analysis, the proportion of ordinals increases, and the proportion of cardinals decreases. However, cardinals are still clearly dominant.

The fact that cardinals dominate our sample suggests that the finding that smaller numbers are used more often than larger numbers, cited in support of a logarithmic representation of numbers (Dehaene & Mehler, 1992), may be expected to hold even if number frequencies were based exclusively on cardinals. This argument is corroborated by the fact that ordinals and nominals, which are less frequent than cardinals, do not skew strongly toward lower magnitudes in our data, and so are unlikely to drive the bias toward smaller numbers by themselves. Another finding that may be expected to replicate in a cardinals‐only sample is that larger numbers are used more approximately than smaller numbers (Rinaldi & Marelli, 2020; Woodin et al., 2024), which may reflect the increasing imprecision of the approximate number system at higher magnitudes (DeWind et al., 2015; Shepard et al., 1975). Indeed, our data demonstrate directly that frequencies of round numbers (i.e., multiples of 5), which are often used approximately (e.g., Krifka, 2009; Solt, Cummins, & Palmović, 2017), increase with magnitude only for pure cardinals and measurements. This is true despite measurements possibly relying less on multiples of 5 for approximations (see Cummins, 2015, Ch. 6). Generally, most of the pure cardinals and measurements in our dataset are round, whereas most of the ordinals and nominals are unround. Of course, this result does not mean people never round ordinals or nominals—there is evidence for roundness being relevant to ordinal numbers (Cummins & Franke, 2021; Isaac & Schindler, 2014; Van der Henst et al., 2002)—but our dataset indicates that rounding is much more common for quantitative uses of numbers, which are more closely tied to the approximate number system.

Regarding approximation, Winter and Marghetis (2023) note that the lexical differentiation of English reveals a strong communicative need to express quantity approximately. English has many lexical items to express approximate quantity, including quantifiers (e.g., “a few,” “several,” “more than,” “most”), range expressions (e.g., “10–15”), nouns (e.g., “a bulk of,” “a heap of”), and number modifiers (e.g., “N‐ish”). Our data suggest that even with the lexical items that afford the most precision—numbers—we so often use them approximately, as round numbers. This is particularly the case for very high numerical magnitudes. In fact, even some ostensibly unround numbers at higher magnitudes are intended as approximations, as a form of numerical hyperbole (see Kao, Wu, Bergen, & Goodman, 2014; Lavric, 2010), such as the use of a large round number *plus one*, as in “a million and one ideas.” Although these hyperbolic numbers are unround, they are based on round numbers, which act as a benchmark (Dehaene, 1997, Ch. 4; Gunasti, 2016), with an added “one” to indicate that the quantity is even larger than an already large amount. Constraining the interpretation of these numbers as imprecise is the fact that they are often unrealistically high in context (e.g., “thousand and one gas lamps”), or quantify things that are difficult to count precisely (e.g., “ideas,” “tadpoles”), at least at the high magnitudes discussed.

People talk and write most often about the things that are most communicatively relevant to them: language users in colder climates discuss ice and snow more frequently (Regier, Carstensen, & Kemp, 2016); English speakers in vision‐based Western cultures use vision‐related words more frequently (Winter, Perlman, & Majid, 2018), and addition‐based language is used more frequently than subtraction‐based language (Winter, Fischer, Scheepers, & Myachykov, 2023), mirroring an addition bias found in behavioral studies (Adams, Converse, Hales, & Klotz, 2021). Our analysis of functions shows that, in COCA, pure cardinals most often count people, while measurements most often quantify money, ordinals most often represent years, and nominals are most frequent in communications numbers and website programming code. Speakers of American English may assign numbers to people, money, and time more often as it is culturally important to quantify these concepts, as when keeping records of population figures, event attendees, financial transactions, stock market values, historical events, text publication dates, and so on.

While previous studies explored large‐scale register differences in number use in English without differentiating between number types (Woodin et al., 2024), or by only looking at cardinal numbers (e.g., Cushion et al., 2017; Maier, 2002), we investigated register differences in the use of number types. The results for speech, fiction, magazines, and newspapers align with the overall results: measurements are the most frequent, followed by pure cardinals, ordinals, and then nominals. In contrast, academic writing contains more ordinals (e.g., years in citations) and nominals (e.g., zip codes in author addresses). This result is broadly consistent with other register studies that found academic writing contains especially distinctive language (e.g., Biber & Gray, 2010; Conrad & Biber, 2009; Kennedy, 1998). This register difference highlights the importance of conducting research on a representative sample across a range of registers if the results are intended to generalize beyond one's sample to, for example, a language variety (e.g., American English) more broadly.

Certain aspects of our findings cannot be expected to generalize beyond modern American English. For a start, nominal numbers may be much less relevant or even absent from non‐industrialized cultures that do not have phone numbers, credit cards, and so on. Regarding ordinals, even if it were true that many cultures often use numbers to represent years, the specific numbers used for this function would depend on the calendrical system used. In our sample, the ordinals in the range 1,000–10,000 are only frequent due to the Gregorian calendar used in the United States beginning 2024 years ago at the time of writing. In comparison, the Republic of China calendar used in Taiwan begins in the Gregorian year 1912, meaning that the Gregorian year 2024 is ROC era 113 (Wilkinson, 2000). As we have discussed (see Section 3.1), there are some issues with COCA's sampling of texts that may limit its generalizability even to other kinds of contemporary American English, like casual conversation. Our results may also be time‐dependent, with, for example, representational formats of numbers known to vary across time (e.g., Berg & Neubauer, 2014; Chrisomalis, 2020; MacQueen, 2010).

In COCA's written subcorpora, different number types are typically represented in different formats (see Chrisomalis, 2020, Ch. 6). Representational format seems strongly prescribed for ordinals and nominals: the overwhelming majority of these numbers are numerals. In comparison, whereas cardinals are more often written as number words in general, representational format is far more varied for this number type, especially for measurements. The increased formatting of measurements as numerals compared to pure cardinals may be because, conventionally in English, units of measurements are permitted to be abbreviated or represented as a symbol when used with numerals (e.g., “100 m”, “$20”) but not number words (e.g., “one hundred meters”, “twenty dollars”). Due to this convention, there may be a greater communicative pressure for writers to use numerals to keep measurements short. Communicative pressure toward concision may also help explain why larger, unround measurements are especially likely to be written as numerals: unround number word expressions increase in length with magnitude (e.g., “one thousand and fifty three” is longer than “one thousand”) as they are formed partly through the concatenation of round number words, while round number word expressions do not necessarily become longer with magnitude (e.g., “one million” is shorter than “one thousand”). Hence, writing unround measurements as numerals yields a greater improvement in concision than for round measurements, especially for larger numbers. Of course, authors do not have total freedom in their written representation of numbers; rather, they can be constrained by the prescriptions of writing style guides. Indeed, regarding round numbers, the Chicago Manual of Style's (2017a) online FAQ section states, “The whole numbers *one* through *one hundred* followed by *hundred*, *thousand*, or *hundred thousand* are usually spelled out,” whereas numerals are prescribed elsewhere. Even so, style guide prescriptions like this often reflect non‐arbitrary concerns of economy, such as avoiding expressions that are overly long.

Given that, as discussed in the introduction, any morphologically unmarked number could in principle be cardinal, ordinal, or nominal, how does a language user know how to interpret a number? As well as cues provided by constructions, like “bus line number 23,” “player number 23” (Wiese, 2004, pp. 270–274), “top *N*,” and “bottom *N*,” our results reveal other probabilistic cues on which language users may rely. Owing to the predominance of cardinals in our dataset, English speakers may expect most numbers to be cardinal, with measurements being slightly more probable than pure cardinals. This expectation may be strengthened if the number is a round number word (e.g., “thousand”), while the predicted probability of the number being ordinal or nominal may increase if it is an unround numeral (e.g., “162”). Collocations may provide an additional clue: if the number is succeeded by a noun (e.g., “people,” “letters”), for example, English speakers may assign a higher probability to the number being cardinal. Another factor, unexplored in this paper, is punctuation: for example, four‐digit numbers with commas (e.g., “2,024”) are very likely to be cardinal, while the same number without commas (e.g., “2024”) is more likely to be interpreted as a year. These factors and more may interact in forming predictions about number use.

The frequency information we present is relevant for developmental research on children's acquisition of number concepts (e.g., Colomé & Noël, 2012; Fischer & Beckey, 1990; Miller et al., 2000). Specifically, we showed how dominant the use of different number types is in adult American English, which provides the input for acquisition of English by children in the United States. Hence, our work raises the possibility that the order in which children acquire cardinals, ordinals, and nominals may be affected by differential exposure to these number uses, rather than acquisition order being associated solely with cognitive difficulty. Our data also provide insight into the contexts in which these number types appear, with pedagogical applications. For example, while explicit instruction on differences between number types may not be common in language teaching, it may be helpful to introduce cardinal, ordinal, and nominal numbers in the contexts in which these numbers are usually found, with corpus‐driven examples that are actually attested in language—such as “5,000 to 8,000 people” and “thirty seven million Americans” for cardinals—rather than artificially constructed sentences. Teaching numbers in their most common contexts first should have more immediate applicability in everyday conversations. This idea is consistent with more general calls from corpus linguists for the most common concepts, established through corpora, to be prioritized in language teaching (e.g., Hunston, 2002; Kaya, Uzun, & Cangır, 2022; Sinclair & Renouf, 1988; see McEnery & Xiao, 2011 for a review).

Overall, this paper shows how numbers are actually used in context in a corpus of American English, uncovering a picture of numerical communication more complex and nuanced than aggregated number frequencies reveal by themselves. These results have important insights for numerical and mathematical cognition, with language serving as a channel through which the approximate number system can be observed and analyzed.

### Open Research Badges

This article has earned Open Data and Open Materials badges. Data and materials are available at https://osf.io/mdtb4/.

## Notes

1 Nominals *can*, however, be based on cardinal or ordinal information (see Tantner, 2009; Wiese, 2004). For example, although the main functions of the building number (3 Granby Street), table number (07), and order reference number (1249682) are to uniquely identify the building, table, and food order, respectively, this naming convention is based on ordinal information, such as the order of buildings on the street, or the order of tables in the restaurant.

2 In the spoken subsamples, all 392 numbers that were transcribed in a mixed format were cardinals (e.g., “$10 million a year,” “between 248 and 254 million people”).

3 Of the remaining concordances that were not straightforwardly cardinal, ordinal, or nominal (not shown in Fig. 1), 50.5% (97 concordances) had a number type that could not be determined, 38.5% (74 concordances) had a nominal meaning that was derived from ordinal information (see Section 3.5), 7.3% (14 concordances) referred to numbers themselves in an abstract, “meta” way, and 3.6% (seven concordances) had a nominal meaning that was derived from cardinal information.

4 Note that the percentages and counts are influenced by our sampling method (see Section 3.4): each subsample contains at least 25% unround numbers and 25% round numbers, provided there were enough numbers of each type to sample. Thus, the absolute patterns we observe are different from those that would result from an entirely random sample. Nevertheless, we can describe relative differences.

5 These statistics should be interpreted as an estimate. First, we do not know for a fact that all the morphologically marked numbers are ordinal: some may also be fractions (e.g., “one tenth”). Second, we are extrapolating from a relatively small dataset (*N* = 2,098) to a very large one (*N* = 9,417,350). Third, the smaller dataset has been filtered by several criteria by which the larger one has not (e.g., numbers that were not straightforwardly cardinal, ordinal, or nominal were excluded; see note 3, Section 4.1; also see Section 3.6 for additional exclusions).
